# It Didn’t Have to be This Way Reflections on the Ethical Justification of the Running Ban in Northern Italy in Response to the 2020 COVID-19 Outbreak

**DOI:** 10.1007/s11673-020-10056-1

**Published:** 2020-11-09

**Authors:** Silvia Camporesi

**Affiliations:** Department of Global Health & Social Medicine, School of Global Affairs, King’s College London, Room 3.10 Bush House NE Wing, 30 Aldwych, London, WC2B 4BG UK

**Keywords:** Public health ethics, COVID-19, Coronavirus, Running ban, Italy, Pandemic

## Abstract

In this paper I discuss the ethical justifiability of the limitation of freedom of movement, in particular of the ban on running outdoors, enforced in Italy as a response to the COVID-19 outbreak in the spring of 2020. I argue that through the lens of public health ethics literature, the ban on running falls short of the criterion of proportionality that public health ethics scholars and international guidelines for the ethical management of infectious disease outbreak recommend for any measure that restricts essential individual freedoms, such as the freedom of movement. The public health ethics framework, however, falls short of explaining the widespread public support that the running ban has had in Italy. I discuss possible factors which could explain the public support for the ban in Italy. Finally, I raise the question of what societal implications the abandonment of the public health ethics framework based on proportionality might have. I conclude that if it is the case, as the history of pandemics teaches us, we will experience further waves of COVID-19 outbreaks, it becomes very important to raise these questions now, with an eye towards informing public health policies for the management of future COVID-19 outbreaks. This discussion should not become politicized along the lines of liberal pro-lockdown/conservative anti-lockdown. Instead, we should reflect on the trade-offs of lockdown policies according to a pluralist framework, in which COVID-19 related deaths are not the only possible value to pursue.

## Introduction

Bioethicists, including myself, have basked in opportunities to write about the myriad ethical issues arising from the pandemic: from ethical criteria on how to allocate scarce life-saving resources to critically ill patients at the peak of the pandemic; to ethical issues arising from the shortage of personal protective equipment for healthcare professionals, to questions of prioritisation and access to experimental treatments. There are, however, other no less important ethical issues arising from public health policies during lockdown, which have implications for the management of future infectious disease outbreaks and which deserve our utmost attention. In this paper I will discuss the ethical justifiability of the limitation of freedom of movement, in particular of the ban on running outdoors, in Italy.

Italy experienced the COVID-19 pandemic in the spring of 2020 ahead of every other country in Europe, and reacted to it with one of the most stringent lockdowns in Europe. Freedom of movement was severely restricted during the peak of the COVID-19 outbreak, from February 23rd to May 5th in Northern Italy.^1^ Police cars patrolled the streets and enforced the lockdown, and movements from a) to b) were allowed only out of “necessity”: for health reasons or to go grocery shopping. A self-declaration stating the purpose and destination of travel was required. All parks and outdoor spaces were closed; public benches were cordoned off. Although some of the international press have reported that running was allowed in Italy during Phase 1 of the lockdown (Hirsch 2020), this was an inaccurate statement, as Italy was the only country in Europe on a par only with Spain, to adopt a ban on outdoor exercise on the basis of a national decree.

Because of a certain degree of decentralization of executive power in the Italian republic, governors of the twenty regions of Italy can decide autonomously on public health matters concerning their region. In Emilia-Romagna, my region^2^ and the second hardest hit by the coronavirus outbreak after Lombardy, the governor Stefano Bonaccini implemented a restrictive running ban ahead of Italian’s central government decree (Ordinanza Regione Emilia Romagna, March 18^th^, 2020). In response to a journalist pressing him on his decision to ban all types of exercise including walking or running alone, Bonaccini responded:

I am ready to personally accompany to one of our intensive care wards those who say they cannot give up jogging, and everything will be clearer. There are women and men who are going through intensive treatments, many of them die. Behind the numbers of those who die every day, there are people. People for me will never be numbers. (Corneo 2020, author’s translation, ¶3)

Is the relationship between the tragedies unfolding in an intensive care ward at the peak of the outbreak, and the ban on running as apparent as Bonaccini would like it to be?

## Arguments in favour and against the ban on running

One argument often offered for the justification of restrictive measures of individual freedoms is grounded in the precautionary principle. According to this argument, in the context of an emergency, public health policies should err on the side of caution, and weaker evidentiary basis (than outside the emergency) may be sufficient to justify restrictive measures. However, the level of evidence required to justify restrictive measures increases as evidence about the pathogen accumulates (Selgelid 2009a). In the context of the COVID-19 pandemic, the evidentiary basis for a stringent lockdown, while it may have been justifiable at the beginning of the pandemic, will not be sufficient in later phases.

According to another argument, those who go for a run enjoy themselves, and fail to embody the civic duty that is required during what has been described as a “war,” where the mobilization of all citizens is needed. I find this argument problematic as it fuels community invigilators, and a climate of suspicion and fear. At the peak of the outbreak in northern Italy, accounts of neighbours insulting runners from their balconies and reporting them to the police were frequently found in the media. In Padova, in the Veneto Region, the third hardest hit region by the coronavirus outbreak, a man running with his dog but not wearing a mask was beaten up by two other men, resulting in multiple fractures and hospitalization (Pietrobelli 2020). Anecdotal evidence of several other cases has been reported (De Vivo 2020).

A third argument is grounded in the additional strains that injuries derived from running may put on health-care systems already nearing or at capacity. This argument appears to be the most convincing of the three. If it is the case that hospitals are nearing capacity (as was the case in Italy at the peak of the outbreak), then there might be a case for a moral duty not to overburden the healthcare system, on the basis of a parallel with the moral duty not to infect others (Harris and Holm 1995). However, this duty is grounded in empirical data. Emerging data from the United Kingdom and other countries show that A&E hospital wards have seen a dramatic decrease of admissions for fears of getting infected and of “overburdening the system,” with clear health-related harms derived by the COVID-pandemic (Neville et al. 2020).

Outdoor exercise is key to maintaining mental health stability and well-being for prolonged periods of lockdown (Lades et al. 2020). The ban also exacerbates existing inequalities as it affects disproportionately those without access to a private outdoor space, in absence of other measures aimed at mitigating those differences. Hence, one could put forward the argument—subject to further empirical scrutiny—that a ban on outdoor exercise is likely to increase the number of mental health problems, such as anxiety and depression, which have already been reported to increase in the Italian population during lockdown, where there were no other measures in place aimed at mitigating those differences (Rossi et al. 2020).^3^

## Proportionality as key guiding principle in public health ethics for the management of infectious disease outbreaks

Public health policies aiming to contain an infectious disease outbreak have as a primary goal controlling the spread of the pathogen. This public health goal needs to be balanced against other socially legitimate goals, first and foremost respecting individual rights (Selgelid [Bibr CR24][Bibr CR22]). Any limitation to individual freedoms derived by such policies would need to abide by the principle of proportionality. The proportionality between the restrictions of individual freedom and their predicted ability to limit the spread of the pathogen is also the key guiding principle outlined by the WHO for the justification of coercive social distancing measures in case of an infectious disease outbreak (World Health Organization 2016). That this principle should be pursued in the in the context of the COVID-19 pandemic has been reiterated by two leading bioethics deliberative bodies, i.e. the Nuffield Council on Bioethics, and the German Ethics Council (Nuffield Council of Bioethics 2020; German Ethics Council 2020).

As freedom of movement is one of the most essential human rights, the justification for coercive social distancing measures needs to be grounded in the expectation that the measures are going to work to limit the spread of the pathogen. Absent this justification, the restriction of individual freedom of exercise outdoor (and, one could add, of movement) rests on fragile grounds.

The question then becomes one of evidence regarding how the pathogen spreads. While there was, and remains, a degree of uncertainty about how the virus can be transmitted, there was sufficient evidence available in March 2020, at the peak of the Italian outbreak, about the modes of transmissions of SARS-cov-2 by the airborne route. We also knew that the novel coronavirus is transmitted by asymptomatic individuals, and that it can stick to surfaces for protracted periods of time (Yang et al. 2020). On the basis of this evidence, and following a precautionary principle, as long as runners adhere to the following rules, they will *not* be contributing to the spread of SARS-cov-2: 1) running alone, 2) maintaining a safety distance of 2 meters from other runners, 3) not wiping one’s sweat or spitting on surfaces which then can be touched by others, 4) wearing a mask while (if) running in the vicinity of other people.

## Beyond the public health ethics framework: Explaining the public opinion’s support for the ban in Italy

Through the lens of public health ethics literature, the ban falls short of the criterion of proportionality that public health ethics scholars and international guidelines for the ethical management of infectious disease outbreak recommend for any measure which is restrictive of essential individual freedoms. The motivation itself of the ban seems to lack the necessary evidentiary basis for a public health intervention in the context of an outbreak of an infectious disease.

Stopping at the public health ethics framework analysis, however, would fall short of understanding the public support that the ban has received in Italy. Here, I would like to put forward a different interpretation which retrieves the concept of the “*fioretto*” (literally, “small flower”) from the forgotten Italian Catholic tradition. The “*fioretto*,” in the Catholic tradition, is a good deed or small sacrifice that somebody takes upon herself as a sign of devotion. “*Fioretto*” was what children were asked to do during Lent (which, ironically, coincided with lockdown this year). As a child growing up atheist but raised Catholic, as all Italians in my generations were raised, during Lent we were asked to give up something we liked, just to please God (“*senza altra ragione che far piacere a Dio*”*)* (Lamendola 2017). According to this interpretation of sacrifice (giving up something we care for only to please god), the ever present, though not explicit, Italian Catholic Volkgeist undercurrent can help us understand the support for the ban. Through this lens, the ban on running can be understood as sacrifice *per se*; sacrifice for the sake of sacrifice. It is in this sense that the public health ethics principle of proportionality will not work, as it is aimed towards a precise objective, i.e. decreasing the spread of the pathogen.

Another factor which could potentially explain in part the public’s support for the ban is the following. Patient 1 in Italy, leading to the outbreak in Lombardy in Northern Italy, was a young 38-year-old marathoner and Ironman athlete from Codogno, south of Milano, named Mattia (Visotti 2020). Mattia spent twenty days in intensive therapy in late February/March, and likely infected his father (who died of COVID-19) and his pregnant wife (who survived). During the days in February in which he was positive and asymptomatic, Mattia turned out to be a super-spreader, as he engaged in social life and in an amateur soccer league, and ran two half marathons in early February (Donadio 2020).

Some preliminary data have been published postulating a possible link between strenuous exercise and severe cases of COVID-19. Matricardi and co-authors (2020) have put forward a “viral auto-inhalation” hypothesis to explain the link, according to which the transient immune system depression found in athletes, combined the increased mobility of SARS-cov-2 down aerial ways facilitated by intense exercise, lead to severe manifestations of COVID-19. The media have picked up on this hypothesis, concluding that running is bad for your health, and fuelling that hatred against runners which we have witnessed in Italy during the Phase 1 of the pandemic (Manzotti 2020).

## Trust and compliance in public health policies

Trägårdh and Özkırımlı (2020) compare the stringent lockdown model of Spain with the lockdown model of Sweden, where social distancing was not enforced, but was left to individuals’ moral responsibility. They write that they were both “convinced that the ‘Swedish model’ could not be exported to countries such as Spain [or Italy, one might add], where levels of social and institutional trust are much lower” (¶8).

This reasoning implies that, had it been left to us Italians to behave responsibly and socially distance ourselves (like it has been asked of the Swedes) we would not have done it. Admittedly, this is an intuitively appealing and possibly entertaining view, and shared by many. However, it should be resisted. While some commentators may have cited Italians’ inability to respect rules, and thus banning individual running under a precautionary principle approach was justified, this reasoning is extremely dangerous. On this ground, individual freedoms can be limited on the basis of a stereotype, which then further reifies the pre-existing stereotype.

The idea that trust can be quantified, and that there are “high trust” and “low trust” societies as conceptualized by American philosopher Francis Fukuyama (1995) has been challenged by social scientists on the basis of a plethora of empirical data in other contexts in healthcare which show that there is no single, simple answer, such as trust, which can explain compliance or not with public health policies (Camporesi et al. 2017). As a matter of fact, the same authors cited above (Trägårdh and Özkırımlı 2020) could not explain, on the basis of their discrimination between low trust societies (i.e. Spain, Italy) and high trust societies (i.e. Sweden), why the Swedish model was not adopted by other Nordic countries, such as Denmark or Norway, that share many of the social fabric characteristics with Sweden.

While enticing, such binary notions of trust (low/high, continuums and gradients, absent/present) are too simplistic and need to be abandoned. As there is not a single, simple explanation behind vaccination resistance, or climate change denial, so there is not a single, simple explanation which can explain compliance with public health measures in the context of an infectious disease outbreak.

## Conclusions

To conclude, through the lens of public health ethics literature, the ban on running falls short of the criterion of proportionality that public health ethics scholars and international guidelines for the ethical management of infectious disease outbreak would recommend for any measure that restricts essential individual freedoms. The public health ethics framework, however, falls short of explaining the widespread public support for the running ban. The relationship between the state of the intensive wards in Northern Italy at the peak of the outbreak, and the necessity of the ban, is not apparent. There is no causal link, direct or indirect, between the two. Adopting Harry Frankfurt’s definition, linking the state of the intensive wards and the ban on running, is a classic example of “bullshit” (Frankfurt 2009).^4^ While this may seem a provocative or an unnecessarily strong statement, it is not my intention. It is instead a sad realization building on key insights from a philosopher of the ways in which our politicians’ and leaders’ statements often contain basic logic mistakes.

I agree with the conclusions by Trägårdh and Özkırımlı (2020) that: “There is no doubt that what we will see—globally, in the wake of the coronavirus crisis—is the return of the state. The question is, what state” (¶16). What are the societal implications of the abandonment of the public health ethics framework based on proportionality and least infringement on individual freedoms? If, as the history of pandemics teaches us (Jones 2020; Lowy 2020) we will experience further waves of COVID-19 outbreaks, it becomes very important to raise these questions now, with an eye towards informing public health policies for the management of future COVID-19 outbreaks. This discussion should not become politicized along the lines of liberal pro lockdown/conservative anti lockdown. Instead, we should endeavour to reflect on the societal trade-offs of lockdown policies within a pluralist framework, in which COVID-19 related deaths are not the only possible value to pursue.

## Footnotes

1 The national lockdown was two weeks shorter than the lockdown in Northern Italy: March 8^th^ to May 5^th^, 2020.

2 I spent the lockdown in Italy. For a personal account of the lockdown in my hometown of Forlì in Emilia-Romagna see: https://aeon.co/essays/a-bioethicist-on-the-hidden-costs-of-lockdown-in-italy

3 An example of such measures would have been: a) keeping parks open; and b) establishing rota i.e defined times of the day at which people of different ages or residential addresses could go running.

4 Frankfurt cited in Casarotti 2020.
